# A Green Approach for Profiling Naphthenic Acids in Produced Water Using Deep Eutectic Solvents–Polypropylene-Supported Thin-Film Microextraction and LC-HRMS

**DOI:** 10.1021/acsomega.6c04230

**Published:** 2026-07-13

**Authors:** William Henrique Slominski, Allyson Leandro Rodrigues dos Santos, Edmar Martendal, Leandro Wang Hantao

**Affiliations:** † Universidade do Estado de Santa Catarina Centro de Ciências Tecnológicas, Departamento de Química, Programa de Pós-Graduação em Química Aplicada, Rua Paulo Malschitzki, 200, Joinville 89219-710, Santa Catarina, Brazil; ‡ 28132 Universidade Estadual de Campinas, Instituto de Química, Rua Monteiro Lobato, 270, Campinas 13083-872, São Paulo, Brazil

## Abstract

Produced water (PW) is a byproduct of the oil industry that contains naphthenic acids (NAs), whose chemical complexity requires robust analytical methods to unequivocally identify the analytes and prioritize reducing environmental impacts by reducing generated waste. This study presents the development of a green method combining deep eutectic solvents (DES) with thin-film liquid-phase microextraction (TF-LPME) for the molecular profiling and quantitative determination of 16 NAs in PW samples using liquid chromatography coupled to high-resolution mass spectrometry (LC-HRMS), namely, a Fourier transform Orbitrap analyzer. Dispersive liquid–liquid microextraction (DLLME) and TF-LPME were compared, and the latter performed better, as DLLME led to the unwanted formation of stable emulsions in real samples. Main extraction conditions, such as desorption solvent, DES volume impregnated on the polypropylene (PP) support, extraction time, DES selection, desorption solvent, volume, and time, were evaluated to determine the optimal conditions for each studied variable. Validation was performed by determining the main method quality parameters. Determination coefficients were all above 0.99, linear ranges from 0.015 μg L^–1^ to 1.50 μg L^–1^, and detection and quantification limits ranging from 0.0045 μg L^–1^ to 0.45 μg L^–1^ were obtained. The average recovery was 87.9%, with intraday precision (*n* = 3) ranging from 8.0 to 16.0% and interday precision (*n* = 6) ranging from 10.2 to 16.0%. Method application determined a total concentration of 3.19 ± 0.30 mg L^–1^ and revealed slightly distinct profiles for the O_2_ class with a double bond equivalent (DBE) of 2 in the three analyzed PW samples, identifying 11 homologous series and differences in aromatic compounds. The method was compared with five other literature methods, demonstrating superior sustainability as evaluated by AGREEprep metrics, along with excellent analytical performance and low detection limits suitable for the NA concentration ranges found in real matrices. Thus, the DES-TF-LPME method emerges as an efficient and sustainable alternative for environmental monitoring in the petroleum industry.

## Graphical abstract

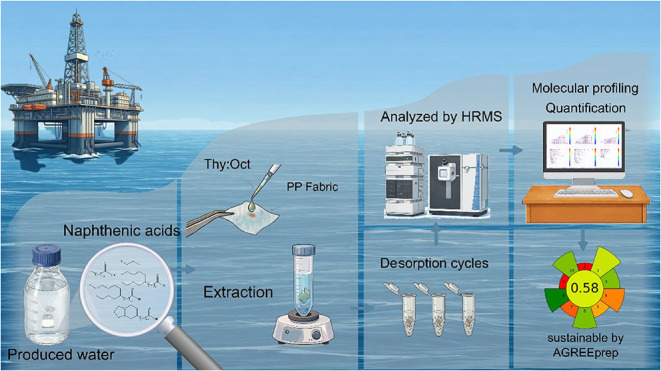

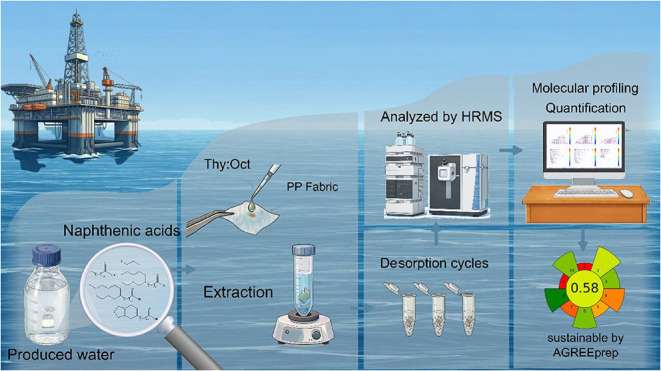

## Introduction

Petroleum is a high-value-added natural resource and a cornerstone of the global market. According to the IEA 2024 Oil Report, annual production is projected to reach 113.8 million barrels per day by 2030.^1^ During offshore oil extraction and production processes, large volumes of saline water, known as produced water (PW), are generated. PW is the largest byproduct of oil extraction, with an annual global production exceeding 15 billion m^3^ and a projected upward trend for the next decade.^2–4^ Produced water (PW) is generated on a large scale and can be reinjected into wells to maintain reservoir pressure and enhance oil recovery, or discharged into the environment, with global discharge volumes estimated in the billions of barrels annually.^5–7^ PW is a complex mixture whose composition varies according to several factors, such as field location, geological formation, and reservoir age.^8,9^ Produced water composition is categorized into inorganic and organic species. The organic fraction consists of polar compounds, such as organic acids and phenols, and less polar compounds, such as polycyclic aromatic hydrocarbons (PAHs) and monoaromatic hydrocarbons, specifically benzene, toluene, ethylbenzene, and xylenes (BTEX).^6,8^

Due to its environmental impact, characterized by biotoxic effects on aquatic life, PW requires treatment prior to its discharge into the environment.^10,11^ In Brazil, PW discharge from offshore platforms is regulated by Resolution No. 393/2007 of the National Council for the Environment (Conselho Nacional do Meio Ambiente – CONAMA), which stipulates that the monthly average for total oil and grease must not exceed 29 mg L^–1^, with a maximum daily limit of 42 mg L^–1^.^12^ Therefore, there is a critical need for reliable analytical methods to assess PWs from both qualitative and quantitative perspectives.

Among the components of PW are naphthenic acids (NAs), which consist of a complex mixture of naturally occurring carboxylic acids.^13^ The class of NAs is highly diverse, comprising aliphatic and cyclic compounds (ranging from one to several rings) as well as aromatic and polycyclic structures (both aromatic and nonaromatic).^8,14^ NAs are described by the general formula C*_n_* H_2*n*+*Z*_O_*x*_, where *n* represents the carbon number, *Z* is a negative integer that indicates hydrogen deficiency, and *x* refers to the oxygen content.^15,16^ The toxicity of NAs is linked to their chemical structure and can cause adverse effects on aquatic biota (including plants, algae, fish, and microorganisms).^17–19^ They are also responsible for refinery equipment corrosion, oil–water emulsion formation, pore plugging, and deposition in processing units.^17–19^ Additionally, the classes and concentrations of NAs vary depending on the origin of the oil. Furthermore, NAs and their salts exhibit pH-dependent solubility in the aqueous phase, influenced by their chemical structures.^20^

The qualitative and quantitative analysis of NAs from PW remains a challenge because PW is complex,^8,21^ which requires an efficient sample preparation prior to instrumental analysis to separate and concentrate the target analytes from the matrix.^3,22,23^ This analysis is crucial due to the economic and environmental impacts of these compounds, which, in addition to causing corrosion in refineries, can precipitate in pipelines, leading to financial losses for the industry.^14^ In this context, several studies have focused on developing analytical methods using various extraction techniques, such as electromembrane extraction (EME),^14^ solid-phase microextraction (SPME),^24^ liquid–liquid extraction (LLE),^25^ and solid-phase extraction (SPE).^13^ The latter two are the most widely used techniques for the extraction and isolation of NAs.^3,8,26^ Our group has explored techniques such as hollow-fiber liquid-phase microextraction (HF-LPME),^8^ automated direct-immersion solid-phase microextraction (DI-SPME),^22^ and vacuum-assisted sorbent extraction (VASE),^27^ aiming to perform both qualitative (characterization) and quantitative analyses of the NAs in PW samples, while developing sustainable methods that eliminate or reduce solvent consumption.

To develop a method capable of simultaneously profiling and quantifying NAs, high-resolution mass spectrometry (HRMS) using analyzers such as Fourier transform ion cyclotron resonance (FT-ICR MS) or Fourier transform orbitrap (FT-Orbitrap MS)^28,29^ is typically required. These techniques have been successfully applied to the characterization of complex matrices using accurate mass measurements of adduct ions and confirmation of isotopic patterns. However, methods involving direct MS analysis can be limited by matrix effects, given the high complexity of the sample.^30^

There is a growing demand for sustainable methods aligned with green chemistry principles, focusing on eco-friendly materials and solvents to minimize the environmental impact of chemical waste. In this context, deep eutectic solvents (DES) have emerged as sustainable alternatives to conventional solvents, garnering significant interest over the last two decades due to their alignment with green chemistry principles. Compared to traditional solvents and even ionic liquids (ILs), DES exhibit superior characteristics, such as higher biodegradability and lower toxicity.^31,32^ DES are defined as products formed through the interaction between a hydrogen bond acceptor (HBA) and a hydrogen bond donor (HBD) and can be composed of two or more components, in a given molar ratio.^33^ The formation of these solvents is driven by hydrogen bonding, van der Waals, and electrostatic interactions, resulting in a significant depression of the melting point compared to the individual pure components.^34^ DES have been used in various extraction techniques for different classes of analytes across various matrices.^34–39^

Direct DES use may hinder solvent recovery due to stable emulsions, favoring the strategy of using DES with supports. Zardo and co-workers exemplified this by supporting hydrophobic DES (HDES) on polypropylene fibers via HF-MMLLE for PAH determination. Optimal results were achieved using levulinic acid:thymol (1:1) for coffee and camphor:thymol (1:1) for tea samples.^40^ Thin-film microextraction (TFME), introduced by Bruheim et al. in 2003, emerges as a promising alternative for employing DES in complex matrices such as produced water (PW). This technique overcomes several limitations, such as the emulsion formation encountered when using DES directly.^41^ The use of DES in TFME enhances analyte interaction by allowing the incorporation of various functional groups onto the film surface and tailoring its properties to specific applications. Additionally, it provides ecological and biodegradable benefits, reducing environmental impacts.^42–44^

This study aimed to develop a method combining the use of DES-supported TFME with LC-HRMS analysis to characterize and determine NAs, which are one of the primary classes of compounds dissolved in PW. To the best of our knowledge, this is the first method developed that applies DES to characterize the chemical profile and quantify NAs in PW, providing a more sustainable alternative aligned with green chemistry objectives. In contrast to former group protocols (HF-LPME, DI-SPME, and VASE), the current TF-LPME method replaces conventional extraction with a DES-based approach, eliminating derivatization. This shift ensures a more sustainable, faster, and low-cost workflow, providing a broader analytical reach for the identification and quantification of NAs.

## Materials and Methods

### Reagents and Materials

A technical mixture of naphthenic acids acquired from Sigma-Aldrich (batch: BCBS3204 V; CAS: 1338-24-5) was used for the preparation of the simulated PW samples. To prepare the DES, thymol (99%), l-menthol (99%), decanoic acid (99%), and octanoic acid (99%) were all obtained from Sigma-Aldrich (St. Louis, MO) and used as received. Other reagents used in this work included LC-MS grade methanol and formic acid, both from Supelco (Bellefonte, PA), while sodium chloride and hydrochloric acid were purchased from Synth (Diadema, SP, Brazil), and polypropylene (PP) fabric (Sigma-Aldrich, St. Louis, MO; product code Z104256) was used as the thin-film support. The NAs used for quantitative analysis and method validation were the following compounds: cyclohexanepentanoic acid, dicyclohexylacetic acid, 1-naphthaleneacetic acid, 2-methyloctadecanoic acid, pentadecanoic acid, benzoic acid, cyclohexanebutyric acid, 9-anthracene carboxylic acid, 2-naphthoic acid, 1-naphthoic acid, 1-adamantane carboxylic acid, myristic acid, cyclohexane carboxylic acid, undecanoic acid, and decanoic acid, all purchased individually in neat form from Sigma-Aldrich (St. Louis, MO).

### Produced Water Samples

Four samples were used in this study. A simulated sample, obtained by diluting the technical mixture of NAs in an aqueous solution containing 10% (w/v) sodium chloride, was used in method development. Additionally, three environmental PW samples were provided by Petrobras. The pH of all PW samples was adjusted to 2 by using a diluted HCl solution.

### Preparation of Deep Eutectic Solvents

The DES were synthesized by combining hydrogen bond acceptors (HBAs), specifically menthol (Men) and thymol (Thy), with hydrogen bond donors (HBDs), including octanoic acid (Oct) and decanoic acid (Dec). The components were weighed into 15 mL flasks according to the specific molar ratios reported in Table S1 and subjected to magnetic stirring at 400 rpm and 80 °C until a homogeneous, colorless liquid was formed. This procedure yielded four distinct DES, which are identified by the abbreviations listed in Table S1.

### Liquid Chromatography Coupled with High-resolution Mass Spectrometry (LC-HRMS)

A Vanquish LC system coupled to an Orbitrap Exploris 120 high-resolution mass spectrometer (Thermo Fisher Scientific, Bremen, Germany) was used throughout the work. Chromatographic separation was achieved on a 50 mm × 2.1 mm i.d. (1.7 μm particle size) Acquity UPLC BEH C18 column (Waters, Milford, MA) maintained at 40 °C. The mobile phase consisted of (A) water with 0.01% (v/v) formic acid and (B) methanol, delivered at a flow rate of 0.3 mL min^–1^. Injection volume was 5 μL. The gradient elution profile started at 20% B, increased linearly to 95% B over 5 min, and held at this condition for 8 min, for a total run time of 13 min. Mass spectrometry was performed using a heated electrospray ionization source in negative mode (H-ESI) with a spray voltage of 3400 V. The capillary and vaporizer temperatures were set at 325 and 370 °C, respectively. The source gas flows were optimized with sheath gas at 50 and auxiliary gas at 10 (arbitrary units). Data were acquired in full scan mode (*m*/*z* 100–1000) at a resolution of 120,000 (FWHM), ensuring high mass accuracy for naphthenic acid identification.

### Fourier Transform Infrared Spectroscopy (FTIR)

The optimal DES, consisting of a 1:1 molar ratio of thymol and octanoic acid (DES 003), was characterized using a Bruker INVENIO-S Fourier transform mid-infrared spectrometer (Ettlingen, KA, Germany) equipped with an attenuated total reflectance (ATR) accessory. Data acquisition was performed via the OPUS software (version 8.2). Infrared spectra were recorded by accumulating 32 scans at a resolution of 4 cm^–1^ across the spectral range of 4000 to 400 cm^–1^, with the resulting data expressed as transmittance versus wavenumber (cm^–1^).

### Sample Preparation

The optimized TF-LPME procedure (Figure 1) for NA extraction was performed as follows: 10 mL of the sample was transferred to a 15 mL centrifuge tube. A PP fabric (1.5 cm × 0.5 cm) was impregnated with 50 μL of a deep eutectic solvent composed of thymol and octanoic acid (1:1 molar ratio) using a micropipette. The fabric was then immersed in the sample, and the system was subjected to vortex agitation for 2 min. After extraction, the PP fabric was removed with tweezers and transferred to a 2 mL microtube for the desorption step. Analytes were desorbed into 300 μL of ethanol by vortexing for 30 s; this desorption process was repeated for three cycles to ensure maximum removal. The three resulting extracts were combined, filtered through a 0.22 μm PTFE membrane into a vial containing a 350-μL insert, and subsequently analyzed by LC-HRMS.

**Figure 1:**
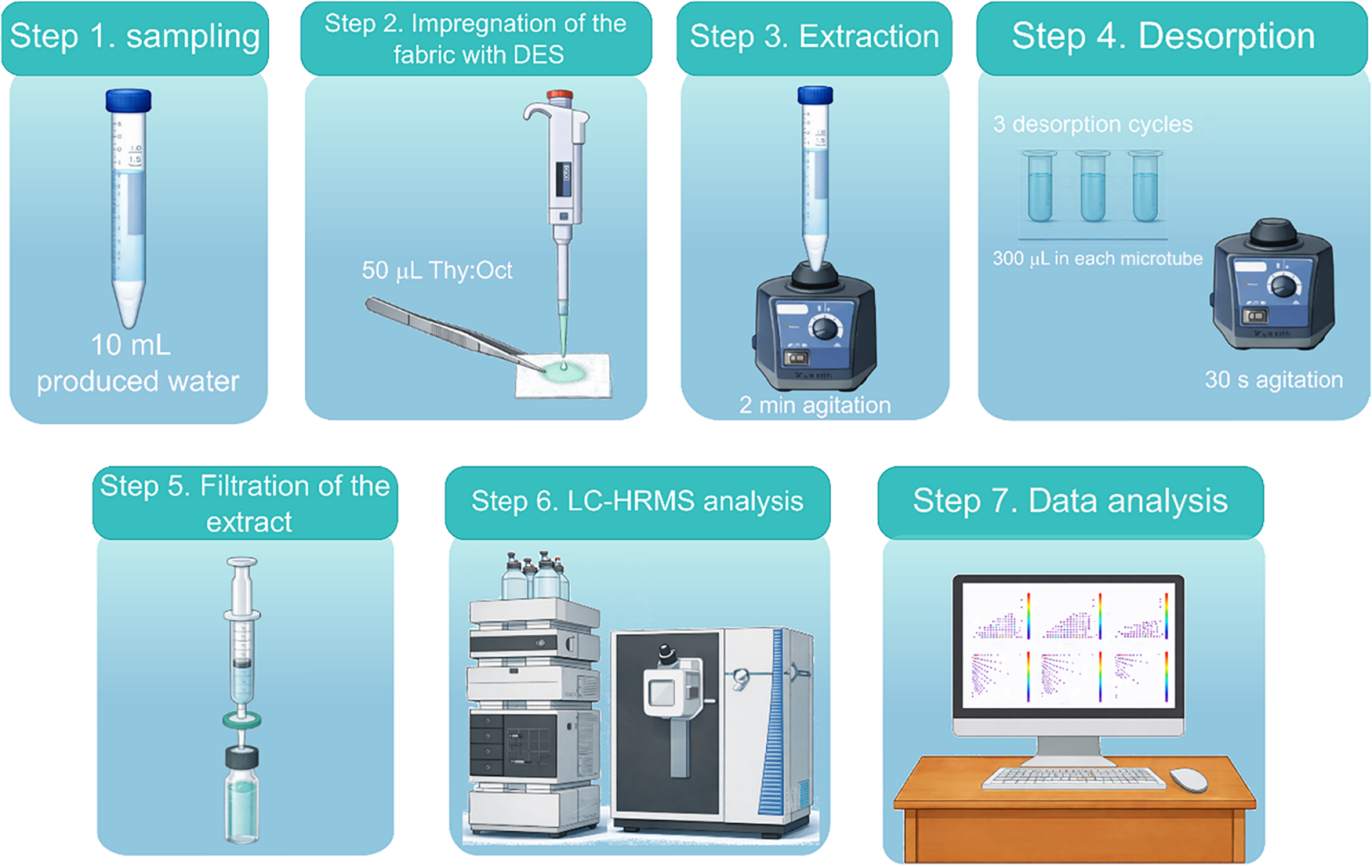
Experimental workflow for the analysis of produced water: (1) sampling; (2) impregnation of the fabric phase with DES 003 (Thy:Oct); (3) extraction via agitation; (4) desorption cycles; (5) extract filtration; (6) LC-HRMS analysis; and (7) data processing and molecular characterization using petroleomic tools. Note: The images were created using Inkscape. The vector elements are in the public domain, and the specific illustrations were generated using Gemini AI (Google).

### Selection of the Extraction Technique

To evaluate the extraction efficiency, dispersive liquid–liquid microextraction (DLLME) and thin-film liquid-phase microextraction (TF-LPME) were compared. Initial assessments were performed using a synthetic sample containing 500 mg L^–1^ of a technical naphthenic acids (NAs) mixture (10% NaCl, pH 2.0), followed by application to a real produced water sample to investigate the impact of matrix components on the extraction performance. For DLLME, 10.00 mL of the sample was transferred to a 15 mL centrifuge tube, followed by the addition of 200 μL of a DES composed of menthol:decanoic acid (1:1). The mixture was vortexed for 1 min and subsequently centrifuged at 9000 rpm for 5 min to induce phase separation. The recovered DES phase (only from the synthetic sample) was collected, transferred to a vial with a 350-μL insert, and analyzed by LC-HRMS. For TF-LPME, a polypropylene (PP) fabric (1.5 cm × 0.5 cm) was impregnated with 100 μL of the DES 002 via a micropipette and then immersed in 10.00 mL of the sample. After vortexing for 1 min, the fabric was removed with tweezers and transferred to a 2 mL microtube. The analytes were desorbed into 300 μL of methanol, and the resulting extract was filtered through a 0.22-μm PTFE membrane before LC-HRMS analysis.

### Optimization of TF-LPME Parameters

To maximize the extraction efficiency and precision of the TF-LPME method for NAs analysis, several experimental parameters were systematically optimized using a synthetic sample as the matrix spiked with 500 mg L^–1^ of the technical mixture. The extraction of O_2_ class species with a double bond equivalent (DBE) of 2 was monitored to evaluate each parameter. The preliminary conditions for these studies were as follows: 10.00 mL of the sample was added to a 15 mL centrifuge tube, and a polypropylene (PP) fabric (1.5 cm × 0.5 cm) was impregnated with the DES 002 using a micropipette. The fabric was then placed directly into the tube containing the sample, followed by vortex agitation. After extraction, the fabric was removed with tweezers and transferred to a 2 mL microtube containing 300 μL of desorption solvent. The microtube was vortexed for 30 s, and the resulting extract was filtered through a 0.22 μm PTFE membrane into a vial (with a 350-μL insert) for LC-HRMS analysis. These conditions were maintained across the subsequent optimization steps.

First, the selection of the desorption solvent (methanol (MeOH), acetonitrile (MeCN), and ethanol (EtOH)) was evaluated to maximize the removal of NA from the PP fabric. The volume of DES 002 deposited onto the PP fabric was studied in three levels: 0 μL (no solvent), 50 μL, and 100 μL. An extraction time profile using a vortex as the agitation system was obtained in the range of 30 to 120 s. The nature of DES was also optimized. Four DES systems were evaluated: menthol:octanoic acid (Men:Oct, DES 001), menthol:decanoic acid (Men:Dec, DES 002), thymol:octanoic acid (Thy:Oct, DES 003), and thymol:decanoic acid (Thy:Dec, DES 004). Different vortex times in the range of 30 to 60 s were studied, as well as the number of desorption cycles of the same PP-TF to verify complete desorption.

### Method’s Quality Parameter Evaluation

For method validation, which focused on the profiling of produced water samples but also aimed at quantitative analysis, the following figures of merit were evaluated in accordance with the recommendations of the Association of Official Analytical Chemists (AOAC): linear range, precision, and accuracy by recovery tests.^45^ Parameters such as the slope, intercept, coefficient of determination, linear range, and limits of detection and quantification were obtained by constructing calibration curves at six levels, performed in triplicate, for both the instrument and the method. Additionally, parameters such as the enrichment factor (EF) were obtained by dividing the slopes of the calibration curves obtained with the method and the instrument.

The first calibration level for each analyte was defined by preliminary tests to determine the lowest area, resulting in acceptable precision and recovery. The concentrations evaluated were LOQ 2×LOQ, 5×LOQ, 10×LOQ, 20×LOQ, 50×LOQ, and 100×LOQ (Tables S2 and S3). All data processing used the extracted ion chromatogram (EIC) with a mass range of 5 ppm for all analytes used in the method’s quality parameter evaluation. The instrumental calibration curve was constructed in acetonitrile and run in the same chromatographic method as that for the samples.

The calibration curve for the method was constructed by spiking a synthetic sample with different concentration levels of the standards described in the Reagents and Materials section. To evaluate the method’s accuracy, recoveries were assessed at LOQ, 10×LOQ, 20×LOQ, and 80×LOQ. To verify the method’s repeatability, intraday precision was evaluated (*n* = 3), and interday precision was also evaluated with a triplicate analysis over two different days, totaling 6 replicates. The levels used to evaluate the intra- and interday precisions were LOQ, 20×LOQ, and 80×LOQ, reported as RSD%.

### Method’s Application

The application of the optimized method was structured into two main stages: (i) analytical validation, conducted by determining figures of merit such as recovery tests and repeatability (evaluated through intraday and interday precision) for quantitative analysis, and (ii) characterization, aimed at profiling NAs in three distinct PW samples, allowing for the identification of variations in both the composition and relative concentration of these compounds.

### Comparison of the Method Using AGREEprep

The method’s sustainability was evaluated using the AGREEprep metric, which focuses specifically on the sample preparation.^46^ This metric consists of 10 criteria aligned with green chemistry principles, each scored from 0 (not met) to 1 (fully met), with the final score displayed at the center of the pictogram. A weight (1 to 5) is assigned to each criterion.^46^ In this study, the weights for criteria 3, 6, 9, and 10 were modified (see Table S4). This adjustment aimed to prioritize sustainable or renewable solvents and materials, ensuring operator safety and high analytical frequency while de-emphasizing sample preparation automation.

The proposed method was compared with five studies from the literature regarding NA analysis in PW, which utilize techniques such as hollow-fiber liquid-phase microextraction (HF-LPME),^8^ solvent-terminated dispersive liquid–liquid microextraction (ST-DLLME),^19^ liquid–liquid extraction (LLE),^25^ electromembrane extraction (EME),^14^ and direct-immersion solid-phase microextraction (DI-SPME).^22^ In addition to the AGREEprep assessment, the methods were compared based on qualitative and quantitative capabilities, limits of detection (LOD), recovery, and precision, covering essential analytical performance parameters alongside the method’s greenness.

### Data Processing

Data acquisition was performed using Xcalibur software (Thermo Scientific, Waltham, MA), while the profiling and characterization of NAs were carried out using Composer 2.0 software (Sierra Analytics, Modesto, CA) based on the average mass spectra. Regarding the data processing parameters, no signal-to-noise (S/N) filtering was applied during the preprocessing.

## Results and Discussion

### Selection of the Extraction Technique

Defining the extraction technique is an essential stage that outlines the entire development of an analytical method, requiring careful attention and critical selection. This importance is even more evident when employing the DES. Since the extractive phase consists of a combination of multiple components, it results in distinct physicochemical properties that lead to different interactions with both target analytes and matrix components. In the case of organic acids, such as NAs, these interactions can lead to the formation of emulsions, which complicate phase separation and collection when DLLME is employed. To circumvent these challenges, the impregnation of solid supports with solvents offers a viable alternative, as the support acts as a physical barrier that prevents emulsion formation. Therefore, properly aligning the extraction technique with the DES properties is a fundamental factor in maximizing the selectivity and minimizing potential matrix interferences.

Consequently, the first stage of this work involved the evaluation and selection of the extraction technique for NA from PW, comparing the DLLME and TF-LPME. Both techniques were evaluated using synthetic samples, considering the specific parameters inherent to each method. To this end, the extraction efficiency of the O_2_ class species with a double bond equivalent (DBE) of 2 was monitored (Figure 2).

**Figure 2:**
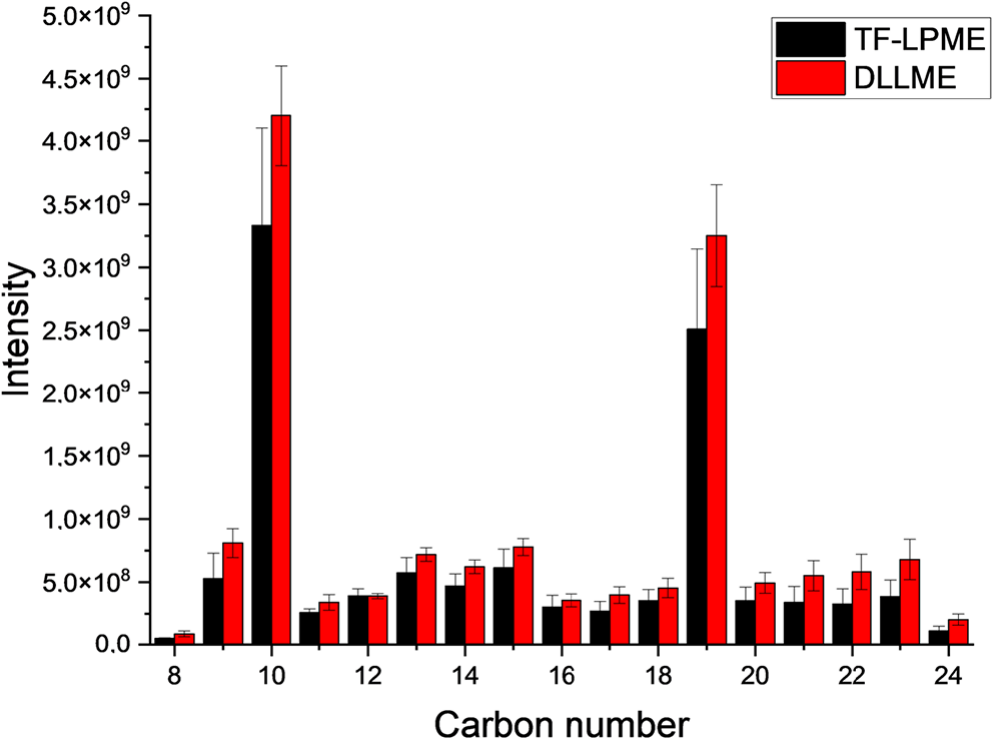
Comparison of acid class extraction (O_2_) with a DBE equal to 2 between the DLLME and TF-LPME techniques in the synthetic sample, in triplicate. Fixed conditions: 500 mg/L of the technical mixture of NAs in the synthetic sample, 1 min of vortexing, and DES 002 (menthol with decanoic acid, in a molar ratio of 1:1), in which for DLLME (A) 200 μL of DES and 5 min of centrifugation at 9000 rpm and for TF-LPME (B) 100 μL of DES in PP tissue (1.5 cm × 0.5 cm), 300 μL of MeOH, and 30 s for the desorption of NAs.

Comparing the extracted profiles and the intensities of each compound, one can conclude that the extraction performances of both techniques are statistically equivalent, with no significant differences in the intensity of individual acids (across different carbon numbers) or the overall profile. However, to determine the most suitable technique for subsequent optimization, the methods were evaluated by using a real PW sample. In this case, DLLME resulted in the formation of a stable emulsion (Figure 3), which hindered the collection of the DES for subsequent analysis and rendered the technique unfeasible. Consequently, TF-LPME was selected for the remainder of the method development, as it proved applicable to the complex matrix of real PW samples.

**Figure 3:**
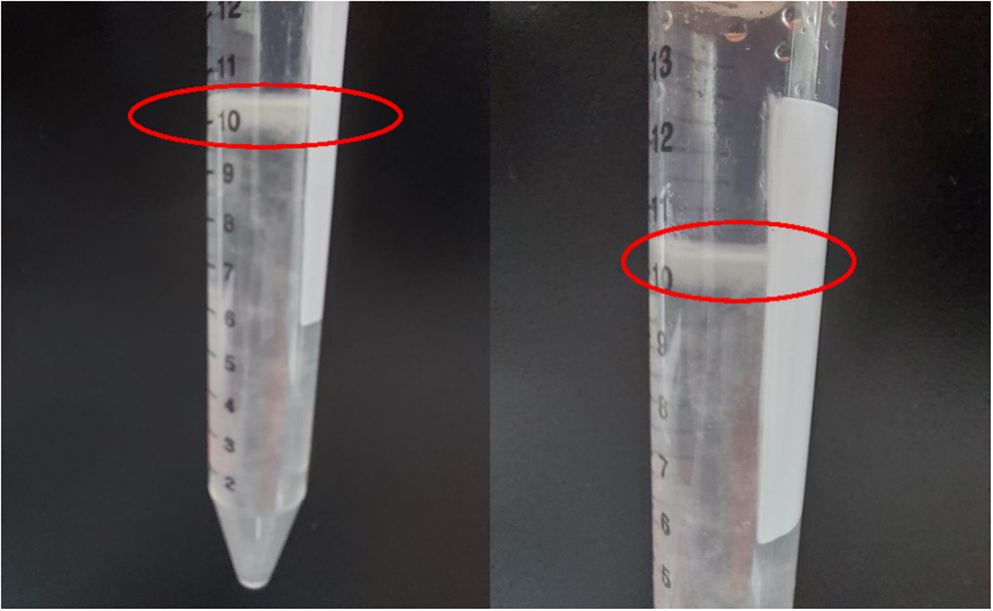
Emulsion resulting from the application of DLLME to the real PW sample.

### Optimization of TF-LPME Parameters

Once the extraction technique was defined, the parameters influencing the performance were evaluated to achieve an optimized method. The first parameter investigated was the desorption solvent for the NAs previously extracted on the PP fabric. The choice of an appropriate solvent must consider not only the solubility of the analytes but also the method’s precision and its environmental impact, such as toxicity. Therefore, three solvents were evaluated: methanol (MeOH), acetonitrile (MeCN), and ethanol (EtOH).

As shown in Figure 4A, no statistical differences were observed in the removal of the NAs from the PP fabric across the different solvents, indicating that any of the three could be effectively employed. To select the most suitable solvent, their safety profiles and toxicological impacts were compared based on their Globally Harmonized System (GHS) pictograms. Methanol is associated with three pictograms (flammability, acute toxicity, and specific target organ toxicity), whereas acetonitrile carries two (flammability and acute toxicity). In contrast, ethanol presents a more favorable safety profile with two pictograms (flammability and eye irritation).^47^ Consequently, ethanol was selected as the desorption solvent because of its lower toxicity and its status as a renewable and sustainable alternative.

**Figure 4:**
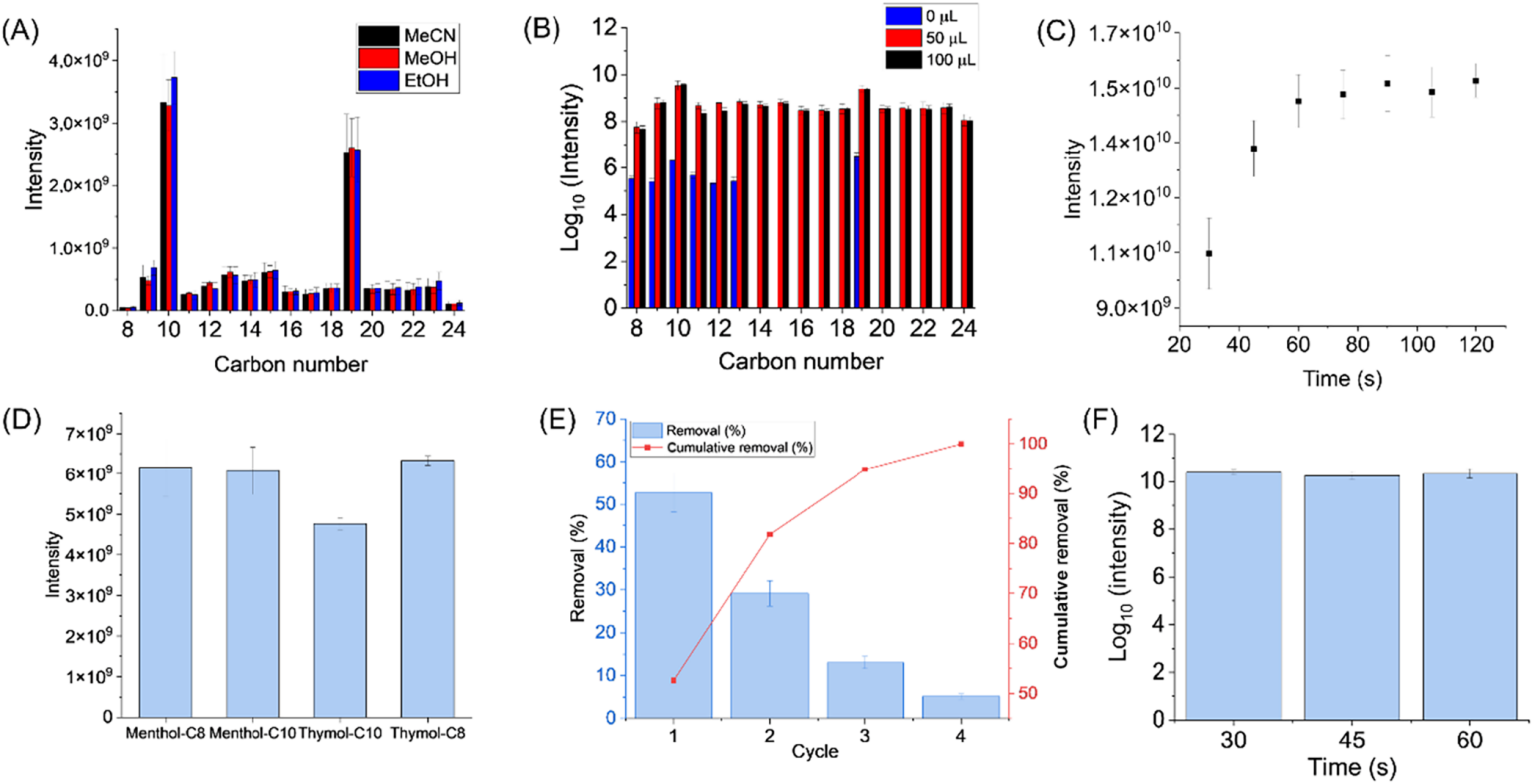
Optimization of TF-LPME parameters for NA extraction was performed in triplicate by monitoring the O_2_ class with DBE = 2. Fixed conditions included the use of PP fabric (1.5 cm× 0.5 cm) and 300 μL of solvent for desorption. The following parameters were investigated: (A) comparison between acetonitrile (MeCN), methanol (MeOH), and ethanol (EtOH) as desorption solvents; (B) evaluation of the DES 002 volume added to the PP fabric, including a neat fabric control (0 μL) with intensities expressed on a log_10_ scale for better visualization; (C) extraction time; (D) selection of the DES composition; (E) number of back-extraction cycles required to ensure exhaustive NA removal; and (F) desorption time, also expressed on a log_10_ scale.

The second parameter evaluated was the volume of the DES 002 impregnated onto the PP fabric. The amount of DES directly influences extraction efficiency and, more importantly, the method precision. It is essential to ensure that the DES covers the entire surface of the fabric to maximize its effective extraction area. However, excessive amounts should be avoided to prevent solvent leaching into the sample matrix, which could impair the extraction of target compounds. In this study, three volumes were evaluated: 50 μL, which was sufficient to cover the surface, and 100 μL, which completely saturated the fabric, and 0 μL (neat PP fabric) to evaluate the support’s extraction capacity. In Figure 4B, the intensities are expressed on a logarithmic scale to facilitate a better visual comparison between the evaluated volumes and the neat PP fabric (0 μL). This highlights a significant increase in extraction efficiency when the PP fabric is impregnated with DES 002, with observed intensities approximately 2 orders of magnitude higher than those obtained with the neat fabric. As shown in Figure 4B, no statistical differences were observed between the two volumes regarding either the extracted profile or the intensity (expressed on a log_10_ scale) of the acids across different carbon numbers. This indicates that 50 μL is the optimal volume for NA extraction, as it provides sufficient surface coverage without compromising stability.

The vortex agitation time was evaluated in the range of 30 to 120 s. This is an essential factor as the equilibration of the analytes between the sample and the extraction phase is time-dependent. The aim was to determine the best conditions taking into consideration the sensitivity, precision, and analytical throughput.

According to Figure 4C, the extraction equilibrium for O_2_ class species with a DBE of 2 was achieved within 60 s, based on the total intensity of the extracted acids. Additionally, it was observed that while the standard deviation remained similar up to 105 s, it was reduced at 120 s. This suggests that the additional time after reaching equilibrium benefits the stability of the system, reducing the minor variation in analyte concentrations between the two phases. Therefore, an extraction time of 120 s was selected to ensure superior precision.

The choice of DES represents a crucial stage, as the solvent composition is a determining factor that can influence both the extraction efficiency (observed through the total intensity of NAs) and, primarily, the precision of the method. Although the evaluated DESs (Men:Oct (DES 001), Men:Dec (DES 002), Thy:Oct (DES 003), and Thy:Dec (DES 004)) share similar chemical natures, subtle variations in their physical properties (such as viscosity and surface tension) can affect their adherence to the PP fabric during vortex agitation. These factors are critical to ensure a stable extractive phase and, consequently, improve the reproducibility of NA extraction from the PW matrix.

As shown in Figure 4D, changes in the DES composition did not lead to a significant increase in extraction efficiency or a shift in the NA profile, expressed as the total intensity of the extracted O_2_ class species with a DBE of 2. An exception was observed for DES 004 composed of thymol and decanoic acid (Thy:Dec), which exhibited the lowest extraction intensity compared to the other three. This similarity in performance is due to the chemical resemblance between the DES components, with the primary differences being the aromaticity of thymol compared to that of menthol (among the HBAs) and a two-carbon difference in the HBDs. However, a significant difference was observed in the precision; the DES formed by thymol and octanoic acid (Thy:Oct, DES 003) presented the best precision. Consequently, Thy:Oct was defined as an optimal solvent for the method.

The removal efficiency of the NAs extracted from the PP fabric was evaluated by investigating the number of cycles required to maximize extraction. The objective was to obtain a comprehensive and representative profile of the NAs for sample characterization, prioritizing qualitative breadth over the strict quantification of the target analytes. Four sequential desorption cycles were performed. As shown in Figure 4E, the cumulative removal reached 95% at the third cycle, and the fourth cycle did not provide a significant increase in the removal percentage. Therefore, it was determined that three desorption cycles are sufficient and optimal for the analysis of NAs in this matrix.

The final parameter evaluated was the desorption time, which refers to the vortex agitation period of the centrifuge microtube. Building upon the back-extraction study, three removal cycles were performed using different desorption times to investigate the relationship between recovery and time, aiming to improve the analytical throughput. The response shown in Figure 4F represents the sum of intensities for the O_2_ class with a DBE of 2, plotted on a logarithmic scale for better visualization. As demonstrated in Figure 4F, the evaluated times had no significant influence on the intensity or NA removal profile. Consequently, a desorption time of 30 s was selected to maximize the analytical frequency of the method.

### DES Characterization

Only the optimized DES was characterized in this work, which was Thy:Oct (1:1). Observing the spectrum for Oct (red) in Figure 5, absorption bands were identified at 2926 and 2857 cm^–1^, characteristic of symmetric and asymmetric C–H stretching, at 1710 cm^–1^ corresponding to C=O stretching, at 1411 cm^–1^ absorption bands for O–H bending, and at 1279 cm^–1^ for C–O stretching vibrations.^48^ For the Thy spectrum (blue), absorption bands were observed at 3173 cm^–1^ characteristic of phenolic O–H stretching vibrations, at 2956 and 2868 cm^–1^ referring to symmetric and asymmetric C–H stretching vibrations, at 1618 cm^–1^ for aromatic C=C stretching vibrations, at 1245 cm^–1^ for C–O stretching vibrations, and at 800 cm^–1^ characteristic of C–H bending for an aromatic ring trisubstituted at positions 1, 2, and 4.^49^ For the DES spectrum, Thy:Oct (purple), absorption bands characteristic of both components are observed, but the formation of the DES can be suggested by the shift of the carbonyl (C=O) stretching bands of Oct to a lower wavenumber, from 1710 to 1705 cm^–1^, suggesting the formation of a hydrogen bond between the carbonyl oxygen and the hydroxyl (−OH) group of thymol, which leads to the weakening of the double bond and reduces its vibration frequency. Additionally, there was a shift in the C–O stretching bond of the phenolic group of thymol to a lower frequency, from 1245 to 1222 cm^–1^, demonstrating that the participation of the thymol oxygen in the hydrogen bond altered its electron density, weakening the C–O bond. These results are corroborated by findings from Jiang and co-workers and Abdallah and collaborators, indicating, therefore, the formation of a DES.^50,51^

**Figure 5:**
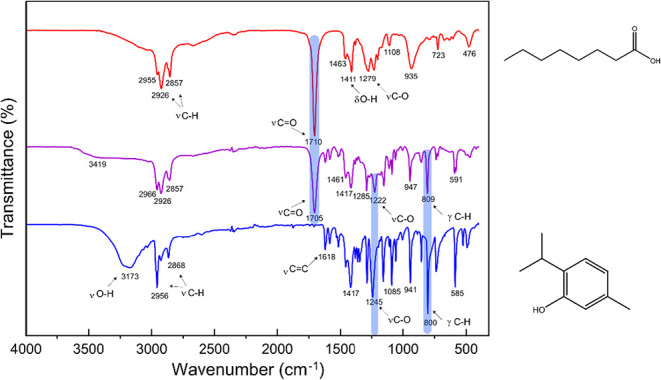
FTIR spectra of pure thymol (Thy, blue), pure octanoic acid (Oct, red), and the deep eutectic solvent (DES 003, purple) formed at a 1:1 molar ratio.

### Method’s Quality Parameter Evaluation

The main figures of merit for the developed method were obtained under the optimized conditions. Parameters such as slope, intercept, coefficient of determination, linear range, and the limits of detection (LOD) and quantification (LOQ) were determined using two sets of calibration curves: instrumental and method-based, performed across six concentration levels (from LOQ to 100×LOQ) in triplicate. A mixture of 16 NAs (see the Materials and Methods section) was used for these experiments. The LOQ (both instrumental and method) was defined as the lowest concentration that yielded acceptable precision (<15), along with satisfactory recovery and analyte identification. Therefore, the LOQ was set as the first point of the calibration curve, and the LOD was calculated as the LOQ divided by 3.3.

Tables S5 and S6 present the linear equations along with the confidence intervals of slopes and intercepts at a 95% confidence level, the coefficient of determination (*R* ^2^), working linear range, LOD, and LOQ. All intercepts were statistically equal to zero at a 95% confidence level. The *R* ^2^ values for all of the analytes were above 0.99. The LOD and LOQ values demonstrate the potential of the method and the LC-FT-Orbitrap MS for the quantitative analysis of complex mixtures in challenging matrices such as PW.

Table 1 presents the enrichment factors, intraday and interday precision, and the concentration determined for each compound in PW 003. The obtained enrichment factors ranged from 2.7 to 8.7. When compared to the maximum theoretical enrichment factor of 11.11 (based on the phase volume ratio of a 10.00 mL sample to 0.9 mL of final extract), most compounds achieved approximately 20% to 50% of the theoretical maximum, with dicyclohexylacetic acid reaching an outstanding 78% (EF = 8.7), indicating an equilibrium-based extraction rather than being exhaustive. These values are considered satisfactory since the primary objective of the extraction was to reduce matrix interference by separating the analytes from the PA complex sample. Furthermore, the high sensitivity of the LC-FT-Orbitrap MS compensated for these recovery rates, as evidenced by the low LOD and LOQ values achieved. Regarding the matrix effect, satisfactory signal suppression ranging from 1.3 to 18.7% was observed, which is a typical phenomenon for complex matrices, such as PW.

**Table 1:** Analytical Performance and Quantification of NAs in the PW Sample^a^

	intraday precision % (*n* = 3)	interday precision % (*n* = 6)	range accepted by AOAC^b^	enrichment factor	concentration in PW (μg L^–1^)
compounds	LOQ	LOQ			
benzoic acid	14.93	12.12	13.2–17.6	4.6	74.6 ± 4.4
cyclohexane carboxylic acid	12.03	12.19	9.6–12.8	3.1	40.7 ± 3.4
cyclohexaneacetic acid	14.84	15.65	19.2–25.6	2.7	578 ± 30
cyclohexanebutyric acid	13.17	13.30	15.0–20.1	3.2	146.5 ± 5.5
1-naphthoic acid	14.57	14.84	15.0–20.1	3.0	1.30 ± 0.55
2-naphthoic acid	13.55	15.39	19.2–25.6	2.9	4.10 ± 0.43
1-adamantane carboxylic acid	13.03	13.87	13.2–17.6	5.2	7.97 ± 0.03
cyclohexanepentanoic acid	15.45	14.12	15.0–20.1	3.1	135 ± 22
undecanoic acid	**13.27**	**13.07**	9.6–12.8	3.0	48.9 ± 4.8
1-naphthaleneacetic acid	**14.91**	10.97	9.6–12.8	3.1	28.1 ± 6.6
myristic acid	12.75	12.70	16.3–21.7	4.7	859 ± 130
dicyclohexylacetic acid	12.85	15.45	15.7–21.0	8.7	58.4 ± 1.1
pentadecanoic acid	12.46	14.18	15.7–21.0	3.7	431 ± 25
9-anthracene carboxylic acid	13.76	14.95	12.8–17.0	3.8	ND
2-methyloctadecanoic acid	15.98	15.96	15.6–20.7	3.5	7.0 ± 1.3
decanoic acid	12.28	12.33	16.3–21.7	5.4	772 ± 63

a Method precision (intra- and inter-day repeatability) assessed by RSD (%), enrichment factor, and concentrations of the 16 compounds used for quantitative analysis of the concentrations determined in the PW sample.

b Value obtained by the Horwitz equation; values above the accepted range are in bold; ND= not detected.

To evaluate the method’s accuracy, recovery tests were performed, and the obtained values were assessed against the maximum limits and intervals established by the AOAC Guidelines. Table S7 presents the accuracy results for the PW sample. As observed in Table S7, for the four evaluated levels (LOQ, 10×LOQ, 20×LOQ, and 80×LOQ), all recovery values fell within the acceptable range defined by the AOAC for their respective concentration levels. The overall average recovery was 87.9%, with RSD values below 15%, demonstrating excellent accuracy and reliability for the analysis of these analytes in that complex matrix. The method’s precision was evaluated through intraday and interday repeatability tests. Intraday precision was assessed by analyzing three replicates within the same day, while interday precision involved the analysis of three replicates across two distinct days. Precision was quantitatively evaluated using the RSD % (see Table S8). The results, summarized in Table 1, were assessed according to the Horwitz equation criteria for intralaboratory tests. Overall, both intraday and interday precision values fell within the acceptable range established by Horwitz, indicating excellent repeatability and reliability for the developed method.

### Application of the Method to Real Produced Water Samples

The developed method was applied to the quantitative analysis of PW samples, using dilutions to ensure that concentrations fell within the linear working range (with a maximum dilution factor of 1:100). The remaining samples were not subjected to quantitative analysis due to their high intrinsic NA concentrations. Applying the method in these cases would require excessive dilutions. The concentrations determined by quantitative analysis in the PW sample are presented in Table 1. The levels in the PW sample ranged from 1.33 to 859 μg L^–1^, with the total sum of quantified compounds resulting in 3.19 ± 0.30 mg L^–1^. For PW samples with higher intrinsic NA levels, the linear working range could be readjusted to avoid excessive dilutions. Nonetheless, the rigorous validation performed at lower concentration levels serves as a proof of concept for the method’s analytical performance.

The qualitative characterization of NAs in the produced water samples was performed by processing the average mass spectra by using Composer 2.0 software. The molecular profiles, presented in Figure 6 through DBE versus carbon number plots and van Krevelen diagrams, revealed a complex and diverse distribution of O_2_ class homologues across all analyzed matrices.

**Figure 6:**
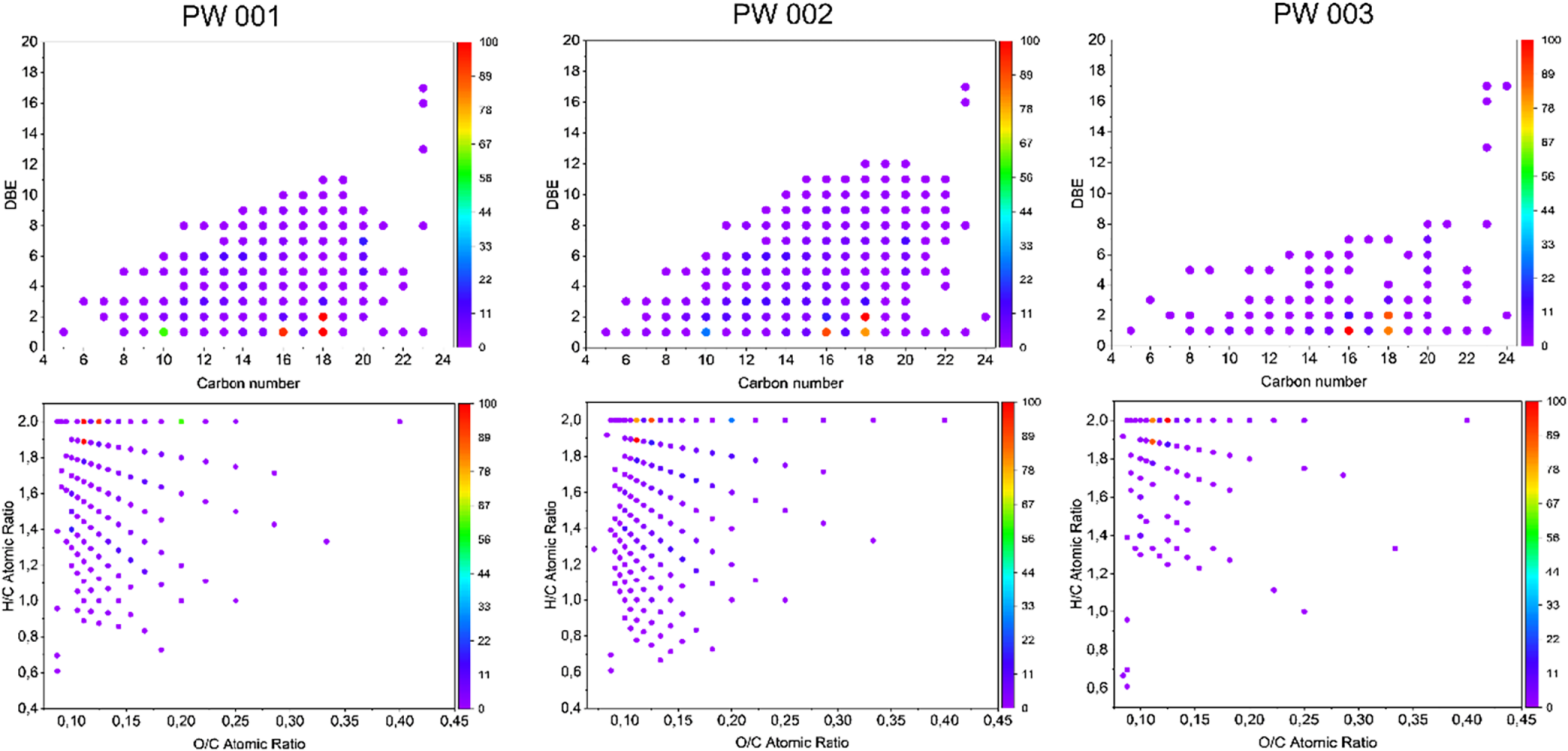
Comprehensive chemical characterization of naphthenic acids (NAs) in produced water samples (PW 001, PW 002, and PW 003). The top row displays DBE versus carbon number plots, while the bottom row shows the corresponding van Krevelen diagrams (H/C vs O/C). Data were processed using Composer 2.0 based on average mass spectra. Color scales indicate the relative abundance of the assigned molecular formulas within the O_2_ class.

The van Krevelen diagram plots H/C vs O/C ratios to classify complex mixtures: aliphatic (2.0), unsaturated or polycyclic (1.0–2.0), or aromatic (<1.0) compounds. O/C ratios indicate oxygenation levels. Values less than 0.1 indicate hydrocarbons, with values between 0.1 and 0.6 identifying oxygenated species such as NAs (carboxylic acids).^52^ Linear trends within the plot reveal homologous series. Sample PW 001 presented the most complex molecular profile among the analyzed waters. The DBE versus carbon number plot shows a higher density of compounds between C14 and C19, with the species of highest relative abundance more concentrated there. This profile indicates a matrix composed mainly of acyclic, monocyclic, and bicyclic acids (DBE 1–3). However, more complex polycyclic structures are also present, as shown in Figure 6, with DBE values reaching up to 11. Analysis of the van Krevelen diagram (Figure 6, bottom) reveals that the PW sample contains 11 homologous series, with compounds concentrated within the H/C atomic ratio range of 1.0 to 2.0. These data corroborate the findings from the DBE versus carbon number plot, indicating that most aliphatic compounds are mono- and polycyclic. Additionally, there is a notable absence of aromatic compounds, which would typically be identified by H/C ratios below 1.0. Consequently, the sample exhibits a high concentration of complex polycyclic structures that, owing to their chemical stability, show significant resistance to biodegradation, thereby imparting a recalcitrant nature to the matrix.^53^ In this context, FT-MS monitoring is essential for accurately assessing the environmental impact of such waters.

Sample PW 002 exhibited a qualitative molecular profile very similar to that observed for PW 001. The DBE versus carbon number plot revealed a homologue distribution centered in the C14–C19 range, with the highest relative abundance in DBE 1–3 structures. Although the visual point density is slightly distinct, the presence of high-DBE compounds and isologous series in the van Krevelen diagram (H/C region 1.2 to 2.0) confirms that the chemical nature (higher presence of polycyclic saturated compounds) and recalcitrant character of the naphthenic acids are common traits of these samples. The most notable difference between samples PW 001 and PW 002 lies in the aromatic compounds, with PW 002 possessing a higher abundance. However, these compounds do not significantly represent the total composition, thus maintaining the overall similarity between the profiles. This suggests that despite minor variations in absolute concentration, the structural diversity and complexity of the extracted O_2_ species follow the same fundamental pattern for both samples. Sample PW 003 exhibited a molecular profile characterized by a homologue distribution primarily concentrated in low-cyclicity structures. In the DBE versus carbon number plot, it is observed that most detected compounds have a DBE between 1 and 3, with an emphasis on carbon series in the C16 to C20 range. The van Krevelen diagram confirms this trend, displaying a point cloud in the higher H/C ratio region, which is indicative of predominantly acyclic and monocyclic acids. Furthermore, it is observed that compared to the previous two samples, PW 003 exhibits a lower molecular diversity, with a reduced number of detected compounds. This composition suggests a matrix rich in compounds with higher water solubility, which is a relevant characteristic for assessing the toxicity and behavior of these acids in produced water disposal.

Overall, the molecular profiles obtained demonstrate that the developed method is capable of mapping matrices with high NA complexity across different PW scenarios. While samples PW 001 and PW 002 revealed greater structural complexity and the recalcitrant nature of polycyclic compounds (slight difference in aromatic compounds), the analysis of PW 003 identified a predominance of low-cyclicity homologues, which are associated with higher risks of acute toxicity. Thus, it can be inferred that the diversity of O_2_-class compounds found across all matrices, combined with TF-LPME and FT-Orbitrap MS, provides a powerful method for rigorous monitoring and environmental impact management within the petroleum industry.

### Method Evaluation and Comparison

The AGREEprep metrics were used to evaluate the method developed in this study and compare it with other works in the literature. In the AGREEprep framework, weights are assigned to 10 criteria; for this work, the weights for criteria 3, 6, 9, and 10 were modified (Table S4).^46^ In addition to this greenness assessment, other essential parameters were compared, as the study’s objective was to develop a sustainable method capable of performing both molecular profiling and quantitative determination of NAs in PW samples.

Table 2 presents the results of this assessment and the comparison between methods, and the scores for each method are shown in Figures S1–S6. Among the evaluated studies, the one utilizing LLE obtained the lowest AGREEprep score. This is attributed to the use of large volumes of dichloromethane (25 mL, three times) for the NA extraction. Additionally, other criteria contributed to the lower scores, such as the reduced analytical frequency, as the technique hinders the preparation of multiple samples per hour, and the requirement for rotary evaporation, which increases the energy consumption per sample. Nevertheless, it is a method developed for the quantitative analysis of eight NAs, exhibiting satisfactory analytical performance.

**Table 2:**
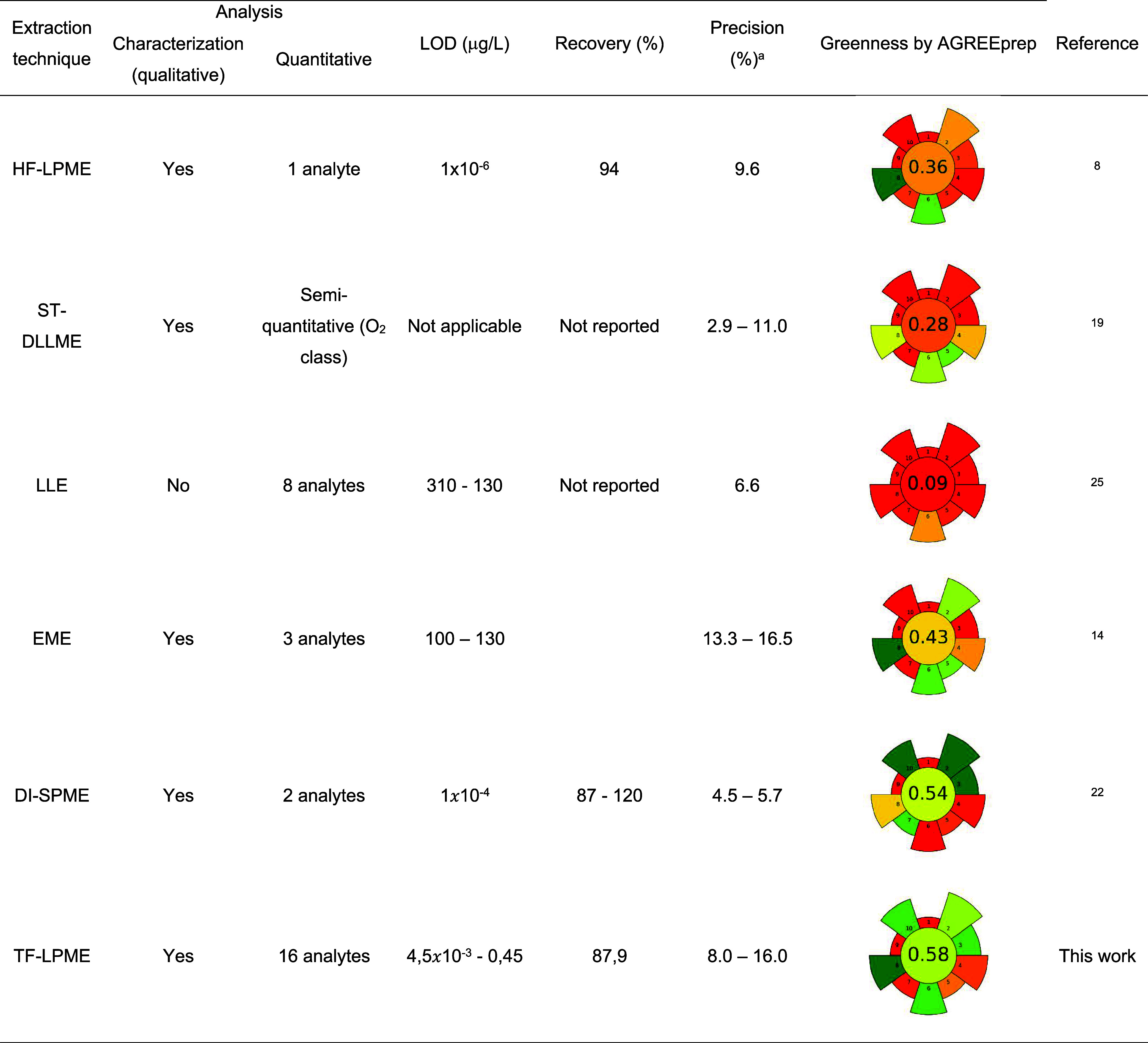
Comparison of Analytical Performance and Sustainability (AGREEprep) of the Developed Method with Reported Extraction Techniques for NA Analysis in PW

a a = data regarding intraday precision.

Methods that achieved scores higher than those of LLE included ST-DLLME and HF-LPME. ST-DLLME was penalized in criteria 2, 3, and 10 due to both the volume and diversity of solvents used (cyclohexane, toluene, acetone, and methanol). The study employing HF-LPME was hindered by its high sample volume, impacting criteria 4 and 5; since PW poses environmental toxicity concerns, larger sample volumes lead to higher waste generation. This factor penalizes all studies, but its impact is more severe for those requiring larger sample volumes. Subsequently, studies using EME and DI-SPME stood out with AGREEprep values indicating greater sustainability. The EME method excelled due to its low sample volume (1 mL) and reduced waste generation, increasing its overall score. DI-SPME strengths were the absence of solvents, minimizing operator risk, and the use of reusable materials (criteria 2, 3, and 10). However, it was penalized for its sample volume, energy consumption resulting from automation, and low analytical frequency, as it allows for the preparation of only one sample per hour.

The method developed in this study achieved the highest score among the evaluated works, performing very closely to the study that utilized automated DI-SPME. The highlights of this approach are the use of a green solvent (DES), a renewable solvent (ethanol) for desorption, and a low-cost polypropylene (PP) support, factors that positively impacted criteria 2, 3, and 10. Other key advantages, in direct comparison with DI-SPME, refer to the lower energy consumption and superior analytical frequency, which enable the preparation of 25 samples per hour. This demonstrates that the proposed method is both sustainable and highly applicable to routine analysis.

Beyond evaluating sustainability and greenness metrics, it is essential to compare the analytical performance of the methods. With the exception of ST-DLLME (semiquantitative analysis) and LLE, where qualitative analysis was not performed, the other methodologies enable molecular profiling and NA determination with an emphasis on quantitative analysis. Notable mentions include the LLE method, developed for 8 NAs, and the present work, which is capable of determining 16 NAs in PW samples. Regarding the limits of detection (LOD), the HF-LPME method showed the lowest value (1.0 × 10^–6^ μg L^–1^), followed by DI-SPME (1.0 × 10^–4^ μg L^–1^) and the present study (0.0045 μg L^–1^ to 0.45 μg L^–1^). These differences do not invalidate the other methods, as the application aims for NA detection and quantification across higher concentration ranges. Finally, parameters such as recovery and precision showed acceptable values in all studies, reinforcing their applicability. Nevertheless, the method developed in this study stands out as an attractive alternative, enabling simultaneous molecular profiling and the determination of 16 NAs while maintaining good performance in the AGREEprep metrics.

## Conclusions

The method developed in this work proved to be an innovative approach, utilizing DES-TF-LPME coupled with LC-FT-Orbitrap MS, for qualitative and quantitative analysis of NAs in PW. The integration of DES as an extraction phase, alongside the PP support, enabled a significant reduction in environmental impact, particularly regarding postanalysis waste generation, as corroborated by the AGREEprep metric, with the method achieving the highest sustainability score among the compared techniques. The analytical performance was excellent, featuring satisfactory detection and quantification limits in the μg L^–1^ range, and was validated with satisfactory recovery and precision. The proposed methodology enables simultaneous qualitative and quantitative analysis, determining the concentration of the 16 studied NAs while obtaining the chemical profile of the samples. The high resolution of the FT-Orbitrap MS (120,000 at *m*/*z* 200) ensures reliable elemental assignment, demonstrating the method’s applicability to the complex PW matrix as a sustainable alternative. This study highlights that the use of alternative solvents (i.e., DES) and low-cost materials (such as PP support) can achieve outstanding analytical performance, even for contaminants in complex matrices.

## Supplementary Material



## Notes

Additional information, such as a table detailing the preparation of the DES; concentration levels used for the instrument and method calibration curves; a description of the criteria used for the AGREEprep assessment; performance metrics for the instrument and method calibration curves; a table showing recoveries at the four evaluated levels; a table of interday and intraday precision; and the reports generated from the studies following evaluation by AGREEprep can be found in supporting information (PDF)
