# Supplementary material for: A Green Approach for Profiling Naphthenic Acids in Produced Water Using Deep Eutectic Solvents–Polypropylene-Supported Thin-Film Microextraction and LC-HRMS
Source: ACS Omega. 2026 Jul 13;11(29):44200–12. doi: 10.1021/acsomega.6c04230 (PMC13425471; doi:10.1021/acsomega.6c04230)
Supplement: Supplementary file 1 [file ao6c04230_si_001.pdf]

Supporting Information

A green approach for profiling naphthenic acids in produced water using deep eutectic solvents-polypropylene-supported thin-film microextraction and LC-HRMS.

William Henrique Slominski[1]; Allyson Leandro Rodrigues dos Santos[2]; Edmar Martendal[1*] and Leandro Wang Hantao[2].

[1]Universidade do Estado de Santa Catarina Centro de Ciências Tecnológicas, Departamento de Química, Programa de Pós-Graduação em Química Aplicada, Rua Paulo Malschitzki, 200, Joinville, 89219-710, Santa Catarina, BR.

[2]Universidade Estadual de Campinas, Instituto de Química, Rua Monteiro Lobato, 270, Campinas, 13083-872, São Paulo, BR.

Corresponding author:

edmar.martendal@udesc.br

Table of Contents

Table S1. DES prepared with their respective combinations of HBA, HHBD, molar ratio, and abbreviation. 3
Table S2. Concentration corresponds to the levels used in the instrumental calibration curve. 4
Table S3. Concentration corresponds to the levels used in the calibration curve of the method. 5
Table S4. Description of the 10 AGREEprep criteria for green sample preparation assessment, showing the default weighting system compared to the specific weights applied in this work. 6
Table S5.Figures of merit obtained from the instrumental calibration curve, including slope, intercept, coefficient of determination (R2), linear range, limit of quantification (LOQ), and limit of detection (LOD). Values in bold are statistically equal to zero with 95 % confidence. 7
Table S6.Figures of merit obtained from the method calibration curve, including slope, intercept, coefficient of determination (R2), linear range, limit of quantification (LOQ), and limit of detection (LOD). Values in bold are statistically equal to zero with 95 % confidence. 8
Table S7. Percentage recoveries obtained for each compound at three concentration levels, with the range accepted by AOAC to the right. (LOQ, 10xLOQ, 20xLOQ and 80xLOQ). 9
Table S8. Method precision in terms of intraday (n=3) and interday (n=6) accuracy assessed at 4 levels (LOQ, 10xLOQ, 20xLOQ and 80xLOQ). 10

Figure S1. Report generated with the respective scores according to each criterion for the reference [8] (HF-LPME) by AGREEprep. 11
Figure S2. Report generated with the respective scores according to each criterion for the reference [14] (ST-DLLME) by AGREEprep. 12
Figure S3.Report generated with the respective scores according to each criterion for the reference [19] (LLE) by AGREEprep. 13
Figure S4. Report generated with the respective scores according to each criterion for the reference [22] (EME) by AGREEprep. 14
Figure S5. Report generated with the respective scores according to each criterion for the reference [25] (DI-SPME) by AGREEprep. 15
Figure S6.Report generated with the respective scores according to each criterion for the method of this study, of the TF-LPME technique by AGREEprep. 16

HBA	HBD	Molar ratio	Abbreviation	reference
Menthol	Octanoic acid	1:1	DES 001	[1,2]
Menthol	Decanoic acid	1:1	DES 002	[1,2]
Thymol	Octanoic acid	1:1	DES 003	[3]
Thmyol	Decanoic acid	1:1	DES 004	[2]

Table S1. DES prepared with their respective combinations of HBA, HHBD, molar ratio, and abbreviation.

Compounds	LOQ	$2x$LOQ	$5x$LOQ	$10x$LOQ	$20x$LOQ	$50x$LOQ	$100x$LOQ
Benzoic acid	1	2	5	10	20	50	100
Cyclohexane carboxylic acid	5	10	25	50	100	250	500
Cyclohexaneacetic acid	0.05	0.1	0.25	0.5	1	2.5	5
Cyclohexanebutyric acid	0.25	0.5	1.3	2.5	5	13	25
1-Naphthoic acid	0.25	0.5	1.3	2.5	5	13	25
2-Naphthoic acid	0.05	0.1	0.25	0.5	1	2.5	5
1-Adamantane carboxylic acid	1	2	5	10	20	50	100
Cyclohexanepentanoic acid	0.25	0.5	1.3	2.5	5	13	25
Undecanoic acid	5	10	25	50	100	250	500
1-Naphthaleneacetic acid	5	10	25	50	100	250	500
Myristic acid	0.25	0.5	1.3	2.5	5	13	25
Dicyclohexylacetic acid	0.5	1	2.5	5	10	25	50
Pentadecanoic acid	0.25	0.5	1.3	2.5	5	13	25
9-Antharacene carboxylic acid	1	2	5	10	20	50	100
2-Methyloctadecanoic acid	0.25	0.5	1.3	2.5	5	13	25
Decanoic acid	0.25	0.5	1.3	2.5	5	13	25

Table S2. Concentration corresponds to the levels used in the instrumental calibration curve.

Compounds	LOQ	$2x$LOQ	$5x$LOQ	$10x$LOQ	$20x$LOQ	$50x$LOQ	$100x$LOQ

Benzoic acid	0.18	0.36	0.90	1.8	3.6	9.00	18
Cyclohexane carboxylic acid	1.5	3.0	7.5	15	30	75.	150
Cyclohexaneacetic acid	0.015	0.030	0.075	0.15	0.30	0.75	1.5
Cyclohexanebutyric acid	0.075	0.15	0.38	0.75	1.5	3.8	7.5
1-Naphthoic acid	0.075	0.15	0.37	0.75	1.5	3.8	7.5
2-Naphthoic acid	0.015	0.030	0.08	0.15	0.30	0.75	1.5
1-Adamantane carboxylic acid	0.18	0.36	0.90	1.8	3.6	9.0	18
Cyclohexanepentanoic acid	0.075	0.15	0.38	0.75	1.50	3.8	7.5
Undecanoic acid	1.5	3.0	7.5	15	30	75	150
1-Naphthaleneacetic acid	1.5	3.0	7.5	15	30	75	150
Myristic acid	0.045	0.090	0.22	0.45	0.90	2.3	4.5
Dicyclohexylacetic acid	0.057	0.11	0.28	0.57	1.1	2.9	5.7
Pentadecanoic acid	0.056	0.11	0.28	0.56	1.1	2.8	5.6
9-Antharacene carboxylic acid	0.22	0.45	1.1	2.2	4.5	11	22
2-Methyloctadecanoic acid	0.060	0.12	0.30	0.60	1.2	3.0	6.0
Decanoic acid	0.045	0.090	0.22	0.45	0.90	2.3	4.5

Table S3. Concentration corresponds to the levels used in the calibration curve of the method.

Criterion	Criterion description	Default weights	Weights used in this work
1	Favor in situ sample preparation	1	1
2	Use safer solvents and reagents	5	5
3	Target sustainable, reusable, and renewable materials	2	3
4	Minimize waste	4	4
5	Minimize sample, chemical and material amounts	2	3
6	Maximize sample throughput	3	4
7	Integrate steps and promote automation	2	2
8	Minimize energy consumption	4	4
9	Choose the greenest possible post-sample preparation configuration for analysis	2	1
10	Ensure safe procedures for the operator	3	4

Table S4. Description of the 10 AGREEprep criteria for green sample preparation assessment, showing the default weighting system compared to the specific weights applied in this work.

Compounds	slope	intercept	R^2	Linear range	LOQ (μg $L^{-1)}$	LOD (μg $L^{-1)}$
Benzoic acid	$1.03.10^4 \pm 9.8.10^2$	**$2.58.10^2$** $\pm 2.6.10^4$	0.99750	1-100	1.0	0.30
Cyclohexane carboxylic acid	$3.39.10^3 \pm 1.5.10^2$	**$2.46.10^4$** $\pm 3.3.10^4$	0.99783	5-500	5.0	1.5
Cyclohexaneacetic acid	$9.39.10^5 \pm 3.7.10^4$	**$1.59.10^6$** $\pm 8.0.10^4$	0.99827	0.05-5	0.05	0.015
Cyclohexanebutyric acid	$3.62.10^4 \pm 1.1.10^3$	**$5.54.10^4$** $\pm 2.3.10^5$	0.99905	0.25-25	0.25	0.076
1-Naphthoic acid	$1.27.10^5 \pm 4.9.10^3$	**$4.75.10^3$** $\pm 5.3.10^4$	0.99833	0.25-25	0.25	0.076
2-Naphthoic acid	$2.16.10^5 \pm 8.3.10^3$	**$-2.17.10^4$** $\pm 9.0.10^4$	0.99838	0.05-5	0.05	0.015
1-Adamantane carboxylic acid	$8.71.10^4 \pm 1.6.10^3$	**$1.50.10^5$** $\pm 3.4.10^5$	0.99963	1-100	1.0	0.30
Cyclohexanepentanoic acid	$3.70.10^4 \pm 1.6.10^3$	**$-4.20.10^4$** $\pm 6.8.10^4$	0.99798	0.25-25	0.25	0.076
Undecanoic acid	$3.76.10^4 \pm 1.0.10^3$	**$-2.91.10^3$** $\pm 1.1.10^4$	0.99922	5-500	5.0	1.5
1-Naphthaleneacetic acid	$2.99.10^3 \pm 1.2.10^2$	**$-2.25.10^4$** $\pm 2.8.10^4$	0.99856	5-500	5.0	1.5
Myristic acid	$4.85.10^5 \pm 1.4.10^4$	**$1.46.10^5$** $\pm 3.0.10^4$	0.99912	0.25-25	0.25	0.076
Dicyclohexylacetic acid	$1.53.10^5 \pm 3.4.10^3$	**$-1.41.10^4$** $\pm 3.7.10^4$	0.99946	0.5-50	0.50	0.15
Pentadecanoic acid	$2.71.10^4 \pm 1.0.10^3$	**$2.78.10^5$** $\pm 2.2.10^4$	0.99839	0.25-50	0.25	0.076
9-Antharacene carboxylic acid	$4.54.10^5 \pm 1.8.10^4$	**$2.70.10^5$** $\pm 2.8.10^5$	0.99819	1-100	1.0	0.30
2-Methyloctadecanoic acid	$3.50.10^5 \pm 9.0.10^3$	**$-3.08.10^5$** $\pm 3.9.10^5$	0.99927	0.25-25	0.25	0.076
Decanoic acid	$2.77.10^4 \pm 1.1.10^3$	**$1.67.10^5$** $\pm 2.6.10^5$	0.99861	0.25-25	0.25	0.076

Table S5.Figures of merit obtained from the instrumental calibration curve, including slope, intercept, coefficient of determination (R2), linear range, limit of quantification (LOQ), and limit of detection (LOD). Values in bold are statistically equal to zero with 95 % confidence.

Compounds	slope	intercept	R^2	Linear range	LOQ (μg L^{-1})	LOD (μg L^{-1})
Benzoic acid	$4.77.10^4$ ± $1.3.10^2$	**$1.35.10^3$** ± $1.0.10^4$	0.99917	0.06 – 6.0	0.18	0.055
Cyclohexane carboxylic acid	$1.04.10^4$ ± $3.0.10^2$	**$-2.32.10^3$** ± $2.9.10^4$	0.99909	1.50 – 150	1.5	0.45
Cyclohexaneacetic acid	$2.50.10^6$ ± 1.4. $.10^5$	**$-1.28.10^4$** ± $8.8.10^4$	0.99678	0.015 – 1.5	0.015	0.005
Cyclohexanebutyric acid	$1.17.10^5$ ± $4.0.10^3$	**$1.46.10^3$** ± $1.3.10^4$	0.99873	0.075 – 7.5	0.075	0.023
1-Naphthoic acid	$3.75.10^5$ ± $1.7.10^4$	**$-1.80.10^4$** ± $5.6.10^4$	0.99770	0.075 – 7.5	0.075	0.023
2-Naphthoic acid	$6.20.10^5$ ± $2.1.10^4$	**$-3.44.10^3$** ± $1.4.10^4$	0.99868	0.015 – 1.5	0.015	0.005
1-Adamantane carboxylic acid	$4.62.10^5$ ± 1.4E.10^4	**$4.47.10^4$** ± $1.1.10^5$	0.99892	0.06 – 6.0	0.18	0.055
Cyclohexanepentanoic acid	$1.12.10^5$ ± $2.8.10^3$	**$1.39.10^3$** ± $9.0.10^3$	0.99932	0.075 – 7.5	0.075	0.023
Undecanoic acid	$1.09.10^5$ ± $3.5.10^3$	**$1.46.10^5$** ± $2.3.10^5$	0.99886	1.50 – 150	1.5	0.45
1-Naphthaleneacetic acid	$8.41.10^3$ ± $3.0.10^2$	**$1.70.10^3$** ± $1.9.10^4$	0.99859	1.50 – 150.	1.5	0.45
Myristic acid	$2.20.10^6$ ± $1.1.10^5$	**$1.63.10^5$** ± $2.1.10^5$	0.99736	0.015 – 1.5	0.045	0.014
Dicyclohexylacetic acid	$1.32.10^6$ ± 5.3. $.10^4$	**$1.62.10^4$** ± $1.3.10^5$	0.99824	0.019 – 1.9	0.057	0.017
Pentadecanoic acid	$1.00.10^5$ ± 4.6. $.10^3$	**$4.39.10^3$** ± $1.1.10^4$	0.99765	0.019 – 1.9	0.056	0.017
9-Antharacene carboxylic acid	$1.68.10^6$ ± $8.4.10^4$	**$2.19.10^5$** ± 2.3. $.10^5$	0.99727	0.075 – 7.5	0.22	0.068
2-Methyloctadecanoic acid	$1.23.10^6$ ± 5.8. $.10^4$	**$-5.81.10^3$** ± $1.5.10^5$	0.99757	0.060 – 6.0	0.060	0.018
Decanoic acid	$1.50.10^5$ ± 7.2. $.10^3$	**$-1.24.10^3$** ± $1.4.10^4$	0.99743	0.015 – 1.5	0.045	0.014

Table S6.Figures of merit obtained from the method calibration curve, including slope, intercept, coefficient of determination (R^2), linear range, limit of quantification (LOQ), and limit of detection (LOD). Values in bold are statistically equal to zero with 95 % confidence.

Compounds	LOQ	Range accepted by AOAC	10xLOQ	Range accepted by AOAC	20xLOQ	Range accepted by AOAC	80xLOQ	Range accepted by AOAC
Benzoic acid	74.4 ± 11.1	40 - 120	103.0 ± 12.7	40 - 120	94.6 ± 9.9	40 - 120	96.9 ± 10.0	40 - 120
Cyclohexane carboxylic acid	82.8 ± 10.0	40 - 120	92.4 ± 10.9	60 - 115	94.9 ± 11.7	60 - 115	102.7 ± 12.2	80 - 110
Cyclohexaneacetic acid	66.8 ± 9.9	40 - 120	88.1 ± 12.9	40 - 120	90.8 ± 9.3	40 - 120	98.8 ± 9.8	40 - 120
Cyclohexanebutyric acid	75.1 ± 9.8	40 - 120	87.6 ± 9.6	40 - 120	89.7 ± 9.5	40 - 120	102.4 ± 10.4	60 - 115
1-Naphthoic acid	107.5 ± 15.8	40 - 120	83.1 ± 10.4	40 - 120	87.9 ± 11.3	40 - 120	87.7 ± 10.8	60 - 115
2-Naphthoic acid	64.7 ± 8.7	40 - 120	91.3 ± 11.8	40 - 120	93.4 ± 11.7	40 - 120	86.5 ± 10.7	40 - 120
1-Adamantane carboxylic acid	80.8 ± 10.6	40 - 120	107.0 ± 13.3	40 - 120	84.5 ± 10.6	60 - 115	93.2 ± 9.9	60 - 115
Cyclohexanepentanoic acid	85.4 ± 13.2	40 - 120	91.7 ± 9.2	40 - 120	92.0 ± 8.8	40 - 120	90.2 ± 8.7	80 - 110
Undecanoic acid	62.8 ± 8.4	40 - 120	102.5 ± 12.8	40 - 120	90.9 ± 10.8	40 - 120	85.2 ± 7.3	80 - 110
1-Naphthaleneacetic acid	58.8 ± 8.8	40 - 120	93.4 ± 12.2	60 - 115	84.4 ± 12.3	60 - 115	93.1 ± 12.6	80 - 110
Myristic acid	57.7 ± 7.4	40 - 120	85.1 ± 10.9	40 - 120	99.2 ± 12.7	40 - 120	88.5 ± 9.8	60 - 115
Dicyclohexylacetic acid	82.4 ± 10.0	40 - 120	94.6 ± 13.0	40 - 120	103.7 ± 10.4	40 - 120	85.7 ± 10.8	60 - 115
Pentadecanoic acid	93.5 ± 11.8	40 - 120	89.0 ± 10.4	40 - 120	87.6 ± 9.8	40 - 120	86.3 ± 10.2	60 - 115
9-Antharacene carboxylic acid	88.9 ± 12.7	40 - 120	97.9 ± 14.4	40 - 120	85.3 ± 11.7	60 - 115	88.1 ± 10.6	60 - 115
2-Methyloctadecanoic acid	86.9 ± 13.6	40 - 120	80.8 ± 9.9	40 - 120	99.9 ± 10.1	40 - 120	91.7 ± 10.4	60 - 115
Decanoic acid	86.8 ± 10.7	40 - 120	103.6 ± 13.2	40 - 120	95.0 ± 11.5	40 - 120	87.3 ± 10.0	40 - 120

Table S7. Percentage recoveries obtained for each compound at three concentration levels, with the range accepted by AOAC to the right. (LOQ, 10xLOQ, 20xLOQ and 80xLOQ).

Compounds	Intra-day precision % (n=3)				Inter-day precision % (n=6)			
	LOQ	10xLOQ	20xLOQ	80xLOQ	LOQ	10xLOQ	20xLOQ	80xLOQ
Benzoic acid	14.9	12.3	10.6	10.3	12.1	13.7	12.9	11.1
Cyclohexane carboxylic acid	12.0	11.6	12.3	12.0	12.2	12.1	10.7	12.2
Cyclohexaneacetic acid	14.8	14.4	10.2	9.9	15.7	13.1	11.1	11.3
Cyclohexanebutyric acid	13.2	11.0	10.4	10.2	13.3	12.3	13.2	11.9
1-Naphthoic acid	14.6	12.7	12.8	12.4	14.8	14.8	14.1	11.3
2-Naphthoic acid	13.5	13.0	12.4	12.1	15.4	12.9	10.3	12.7
1-Adamantane carboxylic acid	13.0	12.5	12.7	10.6	13.9	14.4	10.2	11.0
Cyclohexanepentanoic acid	15.4	10.0	9.6	9.7	14.1	12.9	13.6	11.4
Undecanoic acid	13.3	12.5	11.5	8.0	13.1	13.5	10.3	11.8
1-Naphthaleneacetic acid	14.9	12.8	14.5	13.3	11.0	13.7	11.2	10.2
Myristic acid	12.7	12.9	12.6	11.1	12.7	13.9	12.7	11.5
Dicyclohexylacetic acid	12.9	13.5	10.1	12.5	15.4	13.3	12.3	12.3
Pentadecanoic acid	12.5	11.6	11.3	11.8	14.2	12.5	10.3	11.7
9-Antharacene carboxylic acid	13.8	15.0	13.5	12.0	14.9	15.0	13.5	14.0
2-Methyloctadecanoic acid	16.0	12.3	10.2	10.9	16.0	14.0	10.9	12.0
Decanoic acid	12.3	12.8	12.2	11.4	12.3	13.4	13.6	12.3

Table S8. Method precision in terms of intraday (n=3) and interday (n=6) accuracy assessed at 4 levels (LOQ, 10xLOQ, 20xLOQ and 80xLOQ).

AGREEprep
Analytical Greenness Metric
for Sample Preparation

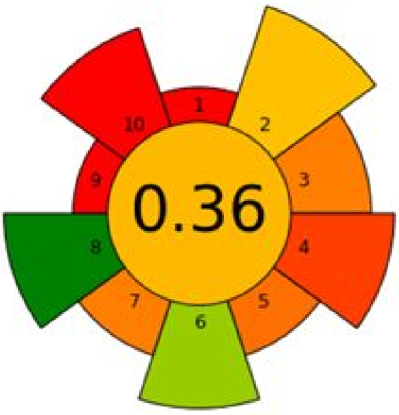

#	Criterion	Score	Weight
1.	**Sample preparation placement**<br>Sample preparation placement: Ex situ	0.0	1
2.	**Hazardous materials**<br>Mass [g] or volume [mL] of problematic materials: 0.73	0.38	5
3.	**Sustainability, renewability, and reusability of materials**<br>25-50% of reagents and materials are sustainable or renewable	0.25	3
4.	**Waste**<br>Mass [g] or volume [mL] of waste: 23	0.12	4
5.	**Size economy of the sample**<br>Mass [g] or volume [mL] of the sample: 22	0.22	2
6.	**Sample throughput**<br>Hourly sample throughput: 20	0.71	4
7.	**Integration and automation**<br>No. of sample prep. steps: 2 steps or fewer; degree if automation: Manual systems	0.25	2
8.	**Energy consumption**<br>Approximate energy consumption per analysis [W]: 2.98	1.0	4
9.	**Post-sample preparation configuration for analysis**<br>Advanced MS with high energy and/or noble gas consumption: ICP-OES, ICP-MS, etc.	0.0	1
10.	**Operator's safety**<br>No. of distinct hazards: 4 or more hazards	0.0	4

Figure S1. Report generated with the respective scores according to each criterion for HF-LPME[4] by AGREEprep.

AGREEprep
Analytical Greenness Metric
for Sample Preparation

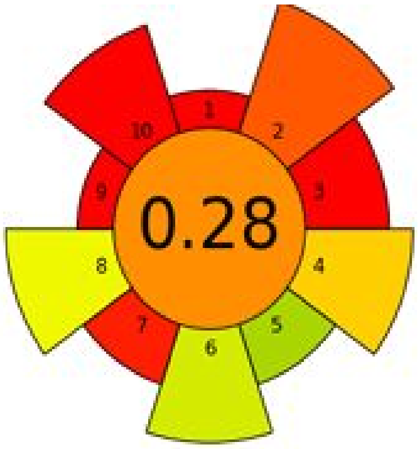

#	Criterion	Score	Weight
1.	**Sample preparation placement**<br>Sample preparation placement: Ex situ	0.0	1
2.	**Hazardous materials**<br>Mass [g] or volume [mL] of problematic materials: 3	0.17	5
3.	**Sustainability, renewability, and reusability of materials**<br>< 25% of reagents and materials are sustainable or renewable, but can only be used ONCE	0.0	3
4.	**Waste**<br>Mass [g] or volume [mL] of waste: 4	0.41	4
5.	**Size economy of the sample**<br>Mass [g] or volume [mL] of the sample: 1	0.67	2
6.	**Sample throughput**<br>Hourly sample throughput: 12	0.58	4
7.	**Integration and automation**<br>No. of sample prep. steps: 5 steps; degree if automation: Manual systems	0.06	2
8.	**Energy consumption**<br>Approximate energy consumption per analysis [W]: 62.5	0.53	4
9.	**Post-sample preparation configuration for analysis**<br>Advanced MS with high energy and/or noble gas consumption: ICP-OES, ICP-MS, etc.	0.0	1
10.	**Operator's safety**<br>No. of distinct hazards: 4 or more hazards	0.0	4

Figure S2. Report generated with the respective scores according to each criterion for ST-DLLME[5] by AGREEprep.

AGREEprep

Analytical Greenness Metric
for Sample Preparation

10/03/2026 11:08:22

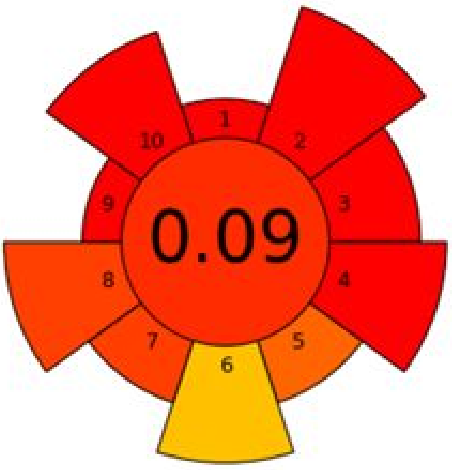

#	Criterion	Score	Weight
1.	**Sample preparation placement**<br>Sample preparation placement: Ex situ	0.0	1
2.	**Hazardous materials**<br>Mass [g] or volume [mL] of problematic materials: 26	0.0	5
3.	**Sustainability, renewability, and reusability of materials**<br>< 25% of reagents and materials are sustainable or renewable, but can only be used ONCE	0.0	3
4.	**Waste**<br>Mass [g] or volume [mL] of waste: 56	0.0	4
5.	**Size economy of the sample**<br>Mass [g] or volume [mL] of the sample: 25	0.2	2
6.	**Sample throughput**<br>Hourly sample throughput: 5	0.38	4
7.	**Integration and automation**<br>No. of sample prep. steps: 4 steps; degree if automation: Manual systems	0.12	2
8.	**Energy consumption**<br>Approximate energy consumption per analysis [W]: 300	0.13	4
9.	**Post-sample preparation configuration for analysis**<br>Advanced MS with high energy and/or noble gas consumption: ICP-OES, ICP-MS, etc.	0.0	1
10.	**Operator's safety**<br>No. of distinct hazards: 4 or more hazards	0.0	4

Figure S3.Report generated with the respective scores according to each criterion for LLE[6] by AGREEprep.

AGREEprep
Analytical Greenness Metric
for Sample Preparation

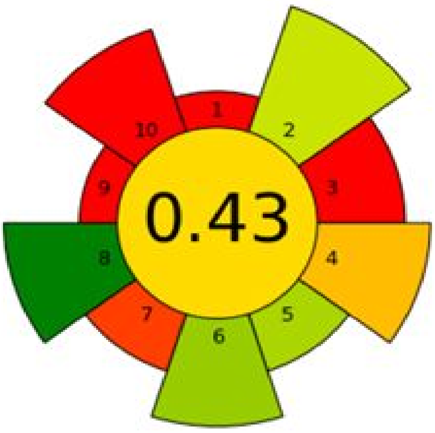

#	Criterion	Score	Weight
1.	**Sample preparation placement**<br>Sample preparation placement: Ex situ	0.0	1
2.	**Hazardous materials**<br>Mass [g] or volume [mL] of problematic materials: 0.15	0.61	5
3.	**Sustainability, renewability, and reusability of materials**<br>< 25% of reagents and materials are sustainable or renewable, but can only be used ONCE	0.0	3
4.	**Waste**<br>Mass [g] or volume [mL] of waste: 5	0.37	4
5.	**Size economy of the sample**<br>Mass [g] or volume [mL] of the sample: 1	0.67	2
6.	**Sample throughput**<br>Hourly sample throughput: 20	0.71	4
7.	**Integration and automation**<br>No. of sample prep. steps: 4 steps; degree if automation: Manual systems	0.12	2
8.	**Energy consumption**<br>Approximate energy consumption per analysis [W]: 2.96	1.0	4
9.	**Post-sample preparation configuration for analysis**<br>Advanced MS with high energy and/or noble gas consumption: ICP-OES, ICP-MS, etc.	0.0	1
10.	**Operator's safety**<br>No. of distinct hazards: 4 or more hazards	0.0	4

Figure S4. Report generated with the respective scores according to each criterion for EME[7] by AGREEprep.

AGREEprep
Analytical Greenness Metric
for Sample Preparation

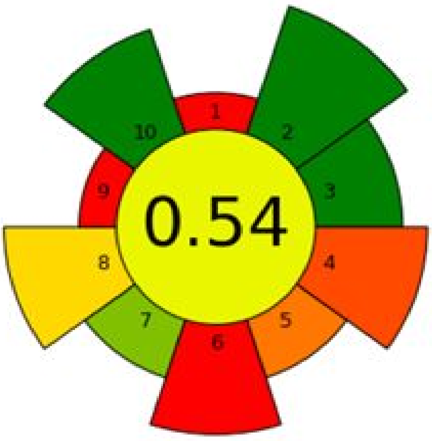

#	Criterion	Score	Weight
1.	**Sample preparation placement**<br>Sample preparation placement: Ex situ	0.0	1
2.	**Hazardous materials**<br>Mass [g] or volume [mL] of problematic materials: 0.005	1.0	5
3.	**Sustainability, renewability, and reusability of materials**<br>Only sustainable and renewable materials are used SEVERAL TIMES	1.0	3
4.	**Waste**<br>Mass [g] or volume [mL] of waste: 20	0.15	4
5.	**Size economy of the sample**<br>Mass [g] or volume [mL] of the sample: 20	0.23	2
6.	**Sample throughput**<br>Hourly sample throughput: 0.92	0.0	4
7.	**Integration and automation**<br>No. of sample prep. steps: 3 steps; degree if automation: Fully automated systems	0.75	2
8.	**Energy consumption**<br>Approximate energy consumption per analysis [W]: 94.2	0.42	4
9.	**Post-sample preparation configuration for analysis**<br>Advanced MS with high energy and/or noble gas consumption: ICP-OES, ICP-MS, etc.	0.0	1
10.	**Operator's safety**<br>No. of distinct hazards: No hazards or no exposure	1.0	4

Figure S5. Report generated with the respective scores according to each criterion for DI-SPME[8] by AGREEprep.

AGREEprep
Analytical Greenness Metric
for Sample Preparation

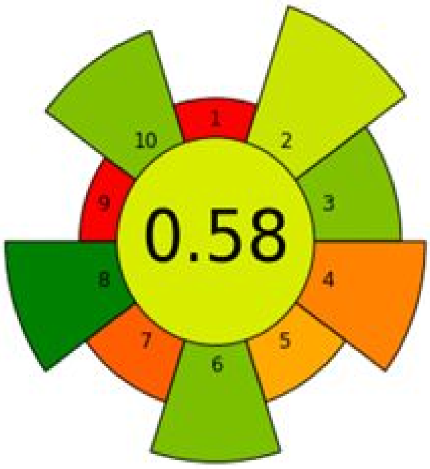

#	Criterion	Score	Weight
1.	**Sample preparation placement**<br>Sample preparation placement: Ex situ	0.0	1
2.	**Hazardous materials**<br>Mass [g] or volume [mL] of problematic materials: 0.15	0.61	5
3.	**Sustainability, renewability, and reusability of materials**<br>> 75% of reagents and materials are sustainable or renewable	0.75	3
4.	**Waste**<br>Mass [g] or volume [mL] of waste: 10	0.26	4
5.	**Size economy of the sample**<br>Mass [g] or volume [mL] of the sample: 10	0.33	2
6.	**Sample throughput**<br>Hourly sample throughput: 25	0.76	4
7.	**Integration and automation**<br>No. of sample prep. steps: 3 steps; degree if automation: Manual systems	0.19	2
8.	**Energy consumption**<br>Approximate energy consumption per analysis [W]: 0.11	1.0	4
9.	**Post-sample preparation configuration for analysis**<br>Advanced MS with high energy and/or noble gas consumption: ICP-OES, ICP-MS, etc.	0.0	1
10.	**Operator's safety**<br>No. of distinct hazards: 1 hazard	0.75	4

Figure S6.Report generated with the respective scores according to each criterion for the method of this study, employing the TF-LPME technique, by AGREEprep.

References

(1) Sereshti, H.; Semnani Jazani, S.; Nouri, N.; Shams, G. Dispersive Liquid–Liquid Microextraction Based on Hydrophobic Deep Eutectic Solvents: Application for Tetracyclines Monitoring in Milk. *Microchemical Journal* **2020**, *158*, 105269. https://doi.org/10.1016/j.microc.2020.105269

(2) Sereshti, H.; Zarei-Hosseinabadi, M.; Soltani, S.; Jamshidi, F.; Shojaee AliAbadi, M. H. Hydrophobic Liquid-Polymer-Based Deep Eutectic Solvent for Extraction and Multi-Residue Analysis of Pesticides in Water Samples. *Microchemical Journal* **2021**, *167*, 106314. https://doi.org/10.1016/j.microc.2021.106314.

(3) Jiang, G.; He, K.; Chen, M.; Yang, L.; Yang, Y.; Tang, T.; Tian, Y. Improvement of Mechanical and Bioactive Properties of Chitosan Films Plasticized with Novel Thymol-Based Deep Eutectic Solvents. *Food Hydrocolloids* **2025**, *158*, 110480. https://doi.org/10.1016/j.foodhyd.2024.110480.

(4) Porto, N. D. A.; Crucello, J.; Facanali, R.; Junior, I. M.; Carvalho, R. M.; Hantao, L. W. Profiling Naphthenic Acids in Produced Water Using Hollow Fiber Liquid-Phase Microextraction Combined with Gas Chromatography Coupled to Fourier Transform Orbitrap Mass Spectrometry. *Journal of Chromatography A* **2021**, *1655*, 462485. https://doi.org/10.1016/j.chroma.2021.462485.

(5) De Araújo, G. L.; Dos Santos, G. F.; Martins, R. O.; Da Silva Lima, G.; Medeiros, I.; De Carvalho, R. M.; Simas, R. C.; Sgobbi, L. F.; Chaves, A. R.; Vaz, B. G. Electromembrane Extraction of Naphthenic Acids in Produced Water Followed by Ultra-High-Resolution Mass Spectrometry Analysis. *J. Am. Soc. Mass Spectrom.* **2022**, *33* (8), 1510–1517. https://doi.org/10.1021/jasms.2c00107.

(6) De Aguiar, D. V. A.; Da Silva, T. A. M.; De Brito, T. P.; Dos Santos, G. F.; De Carvalho, R. M.; Medeiros Júnior, I.; Simas, R. C.; Vaz, B. G. Chemical Characterization by Ultrahigh-Resolution Mass Spectrometry Analysis of Acid-Extractable Organics from Produced Water Extracted by Solvent-Terminated Dispersive Liquid-Liquid Microextraction. *Fuel* **2021**, *306*, 121573. https://doi.org/10.1016/j.fuel.2021.121573.

(7) Crucello, J.; Sampaio, N. Mfm.; Junior, I. M.; Carvalho, R. M.; Gionfriddo, E.; Marriott, P. J.; Hantao, L. W. Automated Method Using Direct-Immersion Solid-Phase Microextraction and on-Fiber Derivatization Coupled with Comprehensive Two-Dimensional Gas Chromatography High-Resolution Mass

Spectrometry for Profiling Naphthenic Acids in Produced Water. *Journal of Chromatography A* **2023**, *1692*, 463844. https://doi.org/10.1016/j.chroma.2023.463844.

(8) Pinheiro, K. M. P.; Sako, A. V. F.; Rodrigues, M. F.; Vaz, B. G.; Medeiros Junior, I.; Carvalho, R. M.; Coltro, W. K. T. Analysis of Naphthenic Acids in Produced Water Samples by Capillary Electrophoresis-mass Spectrometry. *J of Separation Science* **2023**, *46* (19), 2300442. https://doi.org/10.1002/jssc.202300442.
